# Menopause, Reproductive Life, Hormone Replacement Therapy, and Bone Phenotype at Age 60–64 Years: A British Birth Cohort

**DOI:** 10.1210/jc.2016-1828

**Published:** 2016-07-29

**Authors:** D. Kuh, S. Muthuri, R. Cooper, A. Moore, K. Mackinnon, C. Cooper, J. E. Adams, R. Hardy, K. A. Ward

**Affiliations:** Medical Research Council Unit for Lifelong Health and Ageing (D.K., S.M., R.C., A.M., K.M., R.H.), University College London, London WC1B 5JU, United Kingdom; Medical Research Council Lifecourse Epidemiology Unit (C.C., K.A.W.), University of Southampton, Southampton SO16 6YD, United Kingdom; Clinical Radiology and Academic Health Science Centre (J.E.A.), Manchester Royal Infirmary, Central Manchester University Hospital, National Health Service Foundation Trust and University of Manchester, Manchester M13 9PT, United Kingdom; and Medical Research Council Human Nutrition Research (K.A.W.), Cambridge CB1 9NL, United Kingdom

## Abstract

**Context:**

Previous studies of menopausal age and length of reproductive life on bone are limited by retrospective reproductive histories, being cross-sectional, or lacking gold standard bone technologies or information on hormone replacement therapy (HRT) or surgical treatment.

**Objective:**

The objective of the study was to investigate age at menopause, length of reproductive life, and HRT use in relation to volumetric and areal bone mineral density (vBMD, aBMD), bone size, and strength in women aged 60–64 years.

**Design:**

This was a birth cohort study that followed up for 64 years with prospective measures of age at menarche and menopause and monthly HRT histories.

**Setting:**

The study was conducted in England, Scotland, and Wales.

**Participants:**

Participants included 848 women with a known type of menopause and bone measures at 60–64 years.

**Main Outcome Measures:**

Peripheral quantitative computed tomography measurements of the distal radius total and trabecular vBMD were measured. Diaphyseal radius total and medullary cross-sectional area, cortical vBMD, and polar strength strain index (SSI); dual-energy x-ray absorptiometry measurements of aBMD at the lumbar spine and total hip were also measured.

**Results:**

A 10-year increase in age at natural (but not surgical) menopause was associated with 8.2% (95% confidence interval [CI] 1.3%–15.1%, *P* = .02) greater trabecular vBMD and a 6.0% (95% CI 0.51%–11.5%, *P* = .03) greater total vBMD; findings were similar for length of reproductive life. A 10-year difference in HRT use was associated with a 6.0% (95% CI 2.6%–9.3%, *P* < .001) greater polar SSI and a 0.9% (95% CI 0.4%–1.5%, *P* = .001) greater cortical vBMD. These estimates changed little on adjustment. Estimates for aBMD were consistent with those for peripheral quantitative computed tomography.

**Conclusions:**

The positive effects on trabecular vBMD of later natural menopause and longer reproductive life persisted into early old age. HRT use was associated with greater radius cortical vBMD and polar SSI and aBMD.

## Abstract (precis)

Later natural menopause and longer reproductive life, assessed prospectively, were associated with trabecular vBMD in early old age; HRT was associated with cortical vBMD, strength and spine aBMD.

Hip fractures are an important cause of morbidity and mortality in older women, one of the main risk factors for which is low bone mineral density (BMD) (1). Over the last 25 years, earlier timing of natural menopause has been related to lower BMD or subsequent fracture in a number of studies (for example, references 2–6). Some studies have investigated whether the length of reproductive life (2, 7, 8) or oophorectomy and/or hysterectomy (9–12) are associated with lower BMD or fracture, and the findings have been somewhat less consistent. In the UK Million Women Study, postmenopausal women had double the risk of hip fracture compared with premenopausal of the same age; however, in older women, current age had a much greater predictive value than age at menopause (5).

It is essential to take account of hormone replacement therapy (HRT) because patterns of use vary considerably by type and timing of menopause, and HRT is associated with bone health. The Women's Health Initiative trial demonstrated that estrogen plus progestin for healthy women with an intact uterus, and estrogen alone for those with a prior hysterectomy, increased areal BMD (aBMD) and reduced fracture risk (13, 14). However, protection of aBMD and hip fracture starts soon after initiating HRT but does not continue after HRT ceases (15–17). In a study of monozygotic twins comparing pairs in which one twin took HRT and the other did not, HRT was associated with greater volumetric BMD (vBMD) and bone strength at both distal and diaphyseal bone sites (18).

Many previous studies rely on long-term recall of age at menopause, are confounded by age, are cross-sectional or have short follow-up, rely solely on dual-energy x-ray absorptiometry (DXA) or older quantitative bone technologies, or lack information on HRT use and other potentially important confounders or modifiers such as surgical treatment. It also remains unclear whether associations between age at menopause and bone health persist once all women are postmenopausal.

The Medical Research Council National Survey of Health and Development (NSHD), a British birth cohort study with frequent data collections from birth, fills these research gaps because it has prospectively ascertained information on length of reproductive life and type and timing of menopause (19, 20) and HRT use (21) on a large sample of postmenopausal women of the same age with detailed characterization of bone health from DXA and peripheral quantitative computed tomography (pQCT) scans undertaken 10 years after the average age of natural menopause. Use of pQCT allows separate measurement of trabecular and cortical bone. We investigated the timing of the menopause transition, length of reproductive life, and patterns of HRT use in relation to pQCT- and DXA-derived bone outcomes, taking account of current body size, smoking, and socioeconomic circumstances. We hypothesized that earlier age at natural menopause and a shorter length of reproductive life would be negatively associated with trabecular vBMD, whereas HRT use would also be associated with greater cortical bone and bone strength (18, 22).

## Materials and Methods

### Sample

The NSHD is a prospective study of 2547 women and 2815 men followed up 24 times since their birth in a week in March 1946 (23), with a further nine postal questionnaires to women during midlife (19). At age 60–64 years, 2856 study members (of whom 1460 were women) still alive and living at a known address in England, Scotland, or Wales were invited to one of six clinical research facilities (CRFs) across the country; the remaining women were not invited because they had already died (n = 312), were living abroad (n = 258), had previously withdrawn from the study (n = 284), or had been lost to follow-up (n = 233). Of the women invited, 1162 (79.6%) were assessed: 877 women had a clinic visit, with the remaining 285 women opting for a home visit (24). The study received Multi-Centre Research Ethics Committee approval and informed consent was provided by participants.

Of those attending a CRF, 866 women underwent a DXA scan (QDR 4500 Discovery; Hologic Inc), of whom 697 also had a pQCT scan (XCT 2000; Stratec). Details of scan acquisition, data management, cross-calibration, and QA/QC have been described previously (25). Repeat precision was determined in one center and was less than 1% for DXA measurements and for pQCT ranged between 1% and 3%. The bone outcomes were pQCT-derived measures at the radius distal 4% site of total and trabecular vBMD and at the 50% site of diaphysis and medullary cross-sectional area (CSA), cortical vBMD and polar strength strain index (SSI), an estimate of torsional bone strength (26), and DXA-derived measurements of aBMD for lumbar spine (L1-L4) and total hip.

### Timing of menopause

Information on menstrual irregularity, month and year of last menstrual cycle or any operation to remove the uterus or ovaries, and monthly HRT use was obtained from annual postal questionnaires between ages 47 and 54 years (inclusive) with an additional one at 57 years and from face-to-face interviews with trained research nurses at 43, 53, and 60–64 years. Months since birth until periods ceased naturally or because of hysterectomy and bilateral oophorectomy (n = 76), bilateral oophorectomy only (n = 2), hysterectomy and unilateral oophorectomy (n = 21), hysterectomy only (n = 96), or for other reasons were obtained. It was not possible to assign a date of menopause to women who started HRT before the menopause and had not come off HRT for at least a year when giving responses about period regularity and the timing of the last period.

### Age at menarche and length of reproductive life

Age at menarche was obtained from reports of the mother at a medical examination and interview by a school doctor when the study member was aged about 14.5 years. For the 7% of women who had not reached menarche by the time of this examination, retrospective reports obtained from the postal questionnaire at age 48 years were used instead. Length of reproductive life was derived by taking age at menarche from age at natural menopause or hysterectomy (all in months since birth).

### HRT use

From the dates of starting and stopping HRT, we derived an ever-use of HRT (yes vs no), length of HRT use in years, and time since last use (within the last year, 1–5 y ago, more than 5 y ago). Women who provided information on HRT use for at least five of the 10 possible updates were included.

### Other covariables

Height (centimeters) and weight (kilograms) were measured according to a standard protocol at the time of the bone scans and were standardized to give a mean of 0 and a SD of 1. Smoking at age 60–64 years (yes/no) and main occupation (manual vs nonmanual) according to the Registrar General's social class classification were also included as covariates.

### Analysis

Stata version 12.0 was used for all analyses. Regression models used natural logarithms of all bone variables for comparative purposes (27). The coefficients from these models are presented as the mean percentage difference in the bone parameter at 60–64 years between groups for categorical variables or the per-unit change for continuous variables.

We first compared the mean and SDs of the bone outcomes by all of the reproductive and HRT indicators in the maximum available samples. We then fitted three sets of regression models. All were first run unadjusted and then adjusted for height and weight and then for smoking and adult occupation. First, for women with a known age at period cessation, we used nested regression models including type of period cessation, time since period cessation, and the interaction between the two. This allowed us to obtain separate estimates for the percentage difference in the bone outcomes for a 10-year difference in age at natural menopause or age at hysterectomy. We then used similar models to estimate a 10-year difference in length of reproductive life for the natural and surgical menopause groups. Second, for women with a known history of HRT, we obtained estimates for a 10-year difference in length of HRT use and then repeated this analysis for age since last use. Third, for women with a known age at period cessation and history of HRT, we repeated the first set of regression models additionally adjusted for HRT use. Sensitivity analyses were undertaken to see whether any associations between hysterectomy status and bone outcomes differed by oophorectomy status.

## Results

The initial sample comprised 848 women for whom type of menopause was known and who had at least one measure from a DXA or pQCT scan at 60–64 years (Table 1). Of these, 653 women (77%) had a natural menopause and 195 (23%) had a hysterectomy and/or bilateral oophorectomy (henceforth described as hysterectomy) before the menopause. Age at period cessation was known for 709 women; dates were unknown for 134 women because of the timing of their HRT use and for five women who had a hysterectomy. Women who had a hysterectomy were shorter and heavier and had greater vBMD, aBMD, and SSI and strength at 60–64 years than women who had a natural menopause. The mean age of period cessation was 52.0 years for women who had a natural menopause and 44 years 6 months for women who had a hysterectomy. Mean age at menarche, mean length of reproductive life, and HRT use differed by type of menopause.

**Table 1.** Characteristics of the Sample of 848 Women in the Medical Research Council NSHD With at Least One Bone Measure and Known Type of Menopause

Maximum Sample pQCT Measures	Total Sample		Natural Menopause		Hysterectomy and/or Bilateral Oophorectomy		*P* Value
	848^a^ No	Mean (SD)	653 No	Mean (SD)	195 No	Mean (SD)	
Cortical sites: 50% radius							
Diaphysis CSA, mm^2^	681	112.3 (15.8)	523	112.1 (15.5)	158	113.3 (16.8)	.4
Medullary CSA, mm^2^	681	35.2 (12.5)	523	35.6 (12.5)	158	33.8 (12.3)	.1
Polar SSI, mm^3^	682	210.6 (43.1)	524	208.6 (42.2)	158	217.2 (45.4)	.03
Trabecular sites: 4% distal radius							
Distal CSA, mm^2^	674	132.7 (23.9)	518	132.7 (24.5)	156	132.7 (22.0)	>.9
50% radius							
Cortical vBMD, mg/cm^3^	682	1148.2 (39.4)	524	1146.8 (40.2)	158	1152.8 (36.3)	.1
Distal radius (4%)							
Trabecular vBMD, mg/cm^3^	673	171.7 (42.2)	517	169.7 (42.3)	156	178.3 (41.0)	.02
Total vBMD, mg/cm^3^	674	329.3 (70.4)	518	325.5 (70.1)	156	342.0 (69.9)	.01
DXA measures							
Spine L1-L4 aBMD, g/cm^2^	843	.944 (.165)	649	.934 (.164)	194	.976 (.163)	.002
Total hip aBMD, g/cm^2^	839	.869 (.131)	645	.859 (.132)	194	.902 (.123)	<.001
Current body size							
Height, m	848	1.621 (.058)	653	1.624 (.058)	195	1.613 (.057)	.03
Weight, kg	848	72.4 (14.1)	653	71.5 (14.1)	195	75.4 (14.0)	.001
Reproductive measures							
Age at period cessation, y	709	50 y 0 mo	519	52 y 0 mo	190	44 y 6 mo	<.001
		(5 y 9 mo)		(3 y 9 mo)		(6 y 6 mo)	
Age at menarche, y	688	13 y 0 mo	529	13 y 1 mo	159	12 y 10 mo	.04
		(1 y 7 mo)		(1 y 3 mo)		(1 y 4 mo)	
Length of reproductive life	573	37 y 0 mo	418	38 y 10 mo	155	32 y 0 mo	<.001
		(5 y 8 mo)		(3 y 11 mo)		(6 y 5 mo)	
HRT use							
Ever using HRT							<.001
No	277	36.40	247	42.22	30	17.05	
Yes	484	63.60	338	57.78	146	82.95	
Unknown	87		68		19		
Last use of HRT							.5
In the last year	63	13.24	40	12.05	23	15.97	
1–5 y ago	72	15.13	50	15.06	22	15.28	
More than 5 y ago	341	71.64	242	72.89	99	68.75	
Taken HRT but last use unknown	8		6		2		
Total length of HRT use, y							<.001
<1	50	10.57	38	11.48	12	8.45	
1–2	79	16.70	71	21.45	8	5.63	
3–4	78	16.49	54	16.31	24	16.90	
5–6	67	14.16	39	11.78	28	19.72	
7–8	67	14.16	42	12.69	25	17.61	
9–10	53	11.21	42	12.69	11	7.75	
11–12	35	7.40	27	8.16	8	5.63	
≥13	44	9.30	18	5.44	26	18.31	
Unknown length	11		7		4		
Current smoker							
No	760	90.15	585	90.14	175	90.21	>.9
Yes	83	9.85	64	9.86	19	9.79	
Unknown	5	4			1		
Adult social class							
Nonmanual	680	80.28	534	81.90	146	74.87	.03
Manual	167	19.72	118	18.10	49	25.13	
Unknown	1		1		0		

a Sample excludes 13 women whose periods ceased because of medical treatment (eg, chemotherapy) and five women who had been insufficiently followed up to determine menopause type.

### Unadjusted mean differences in bone size, strength and BMD by age at menopause and menarche, length of reproductive life, and HRT use

Neither timing of natural menopause, age at menarche, nor length of natural reproductive life were associated with CSA (diaphyseal or medullary) or SSI (Table 2). Women who had an earlier natural menopause or a later age at menarche had lower mean values of trabecular vBMD, total vBMD, and spine and hip vBMD but not cortical vBMD. Those with a shorter reproductive life had lower mean values of trabecular vBMD, and spine and hip aBMD but not cortical or total vBMD. Age at hysterectomy was not associated with BMD, size, or SSI.

**Table 2.** Mean and SD for Bone Outcomes at 60–64 Years by Menopausal Characteristics

	Diaphysis CSA, mm^2^ Mean (SD)	Medullary CSA, mm^2^ Mean (SD)	Total vBMD, mg/cm^3^ Mean (SD)	Trabecular vBMD, mg/cm^3^ Mean (SD)	Cortical vBMD, mg/cm^3^ Mean (SD)	Polar SSI, mm^3^ Mean (SD)	Spine L1-L4 aBMD, g/cm^3^ Mean (SD)	Hip aBMD, g/cm^3^ Mean (SD)
Age at natural menopause, y								
<45	111.0 (19.6)	36.6 (13.2)	298.2 (73.2)	146.5 (36.2)	1139.9 (39.7)	204.7 (48.4)	0.85 (0.1)	0.83 (0.1)
45–49	109.7 (16.0)	37.1 (14.8)	314.5 (72.4)	167.0 (48.8)	1143.3 (44.1)	199.2 (38.4)	0.89 (0.2)	0.83 (0.1)
50–52	111.9 (14.5)	34.9 (12.1)	320.1 (64.5)	165.8 (40.3)	1149.6 (38.3)	211.8 (43.9)	0.92 (0.2)	0.86 (0.1)
53–55	112.2 (16.3)	36.2 (13.4)	331.7 (75.9)	171.5 (44.1)	1144.1 (41.8)	204.8 (43.2)	0.94 (0.2)	0.88 (0.1)
56–62	112.6 (17)	33.7 (11.1)	321.3 (54.8)	171.9 (35.4)	1149.9 (36.9)	211.9 (45.8)	0.98 (0.2)	0.89 (0.1)
*P* value for trend^a^	.2	.3	.014	.004	.3	.2	<.001	<.001
Age at hysterectomy, y								
<40	115.3 (18)	33.5 (13.1)	337.1 (66.8)	178.1 (37.1)	1150.6 (28.8)	224.7 (47.4)	0.96 (0.2)	0.9 (0.1)
40–44	113.8 (17.8)	35.6 (12.8)	343.9 (60.6)	175.9 (44.3)	1152.4 (36.9)	216.8 (47.3)	0.95 (0.2)	0.9 (0.1)
45–49	112.1 (15.6)	32.1 (12.6)	341.2 (82.2)	179.8 (44.9)	1160 (34.1)	214 (41.5)	0.99 (0.2)	0.9 (0.1)
≥50	111.9 (16.4)	34.5 (11.1)	347.8 (66.6)	179.7 (37.8)	1144.4 (43.1)	213.5 (47.2)	0.99 (0.2)	0.89 (0.1)
*P* value for trend^a^	.5	.7	.7	>.9	.6	.3	.3	>.9
Age at menarche, y								
9–10	111.2 (12.5)	31.4 (9.01)	349.3 (84)	179.9 (40.1)	1158.5 (32.5)	213.2 (34.5)	0.98 (0.1)	0.9 (0.1)
11	113 (17.1)	34 (12.7)	339.2 (69.2)	176.7 (40.6)	1145.7 (44.2)	217.2 (45.1)	0.98 (0.2)	0.9 (0.1)
12	114.4 (16)	36.5 (13.2)	340.5 (74.6)	178.5 (46.1)	1145.9 (39.8)	213.1 (45)	0.95 (0.2)	0.87 (0.1)
13	111.5 (16)	35.7 (12.3)	320.9 (69.2)	169 (41.1)	1148.3 (40)	207.9 (42.5)	0.95 (0.2)	0.86 (0.1)
14	111.5 (14.4)	34.7 (11.9)	318.9 (63.8)	168.4 (34.9)	1151.4 (33)	207.5 (39)	0.92 (0.2)	0.85 (0.1)
15–19	111.9 (17.5)	34.7 (12.8)	336.8 (68.5)	157.7 (39.8)	1150.9 (39.7)	210.9 (38.5)	0.91 (0.2)	0.84 (0.1)
*P* value for trend^a^	.4	.7	.03	.01	.9	.2	.004	<.001
Length of natural reproductive life								
≤35	109.9 (18.7)	37.3 (16.1)	305.1 (70.3)	155.5 (41.9)	1140.9 (44.2)	199.3 (41.3)	0.86 (0.1)	0.8 (0.1)
36–37	109.1 (14)	34.7 (11.2)	324.2 (72.1)	168.6 (46.9)	1152.5 (40.7)	204.6 (41.4)	0.91 (0.2)	0.84 (0.1)
38–39	112 (15.4)	35.5 (11)	323.9 (65.6)	173.4 (38.1)	1151.4 (32.5)	209.7 (46.8)	0.91 (0.2)	0.85 (0.1)
40–41	112.3 (15.9)	35.9 (14.3)	339.3 (69.1)	176.3 (41.1)	1142.9 (44.7)	207.1 (39.3)	0.97 (0.2)	0.9 (0.1)
42–43	116.3 (17.6)	35.9 (11.1)	319.7 (67.8)	165.1 (38.6)	1145.4 (31.6)	220.1 (47)	0.95 (0.2)	0.87 (0.1)
≥44	109.6 (12.5)	34.5 (8.8)	316.6 (63.5)	168.8 (38.4)	1148.2 (39.3)	198 (34.6)	0.98 (0.2)	0.88 (0.1)
*P* value for trend^a^	.07	.9	.1	.02	.4	.07	<.001	<.001
Length of reproductive life (ceased surgically)								
≤35	115.1 (16.4)	34.3 (12.5)	347 (70.3)	180.4 (44.9)	1154.6 (33.7)	221.4 (44.0)	0.97 (0.2)	0.90 (0.1)
36–37	115 (17.5)	38.4 (9.3)	318.4 (80.9)	179.6 (33.6)	1135.8 (35)	214.6 (53.8)	0.99 (0.2)	0.90 (0.1)
38–39	109.5 (14.2)	32.2 (11.5)	344.2 (66.3)	177.7 (45.3)	1151.8 (40.2)	213.7 (39.9)	0.95 (0.2)	0.86 (0.1)
40–41	102.6 (7.2)	24.5 (5)	405.8 (67.2)	204.6 (13.4)	1192.8 (20.9)	184.0 (7.9)	1.09 (0.01)	1.01 (0.1)
42–43	104.9 (17.8)	27.3 (8.3)	391.3 (46.6)	192.7 (21.5)	1169.6 (27.4)	198.4 (43.9)	1.1 (0.2)	0.90 (0.1)
≥44	122.7 (12.2)	42.9 (16.3)	317.1 (89.3)	159.1 (57.8)	1108.9 (77.5)	235.2 (49.7)	0.92 (0.2)	0.88 (0.1)
*P* value for trend	.01	.2	.7	.7	.9	.02	>.9	.7
Length of HRT use, y								
≥13	118.6 (17)	35.4 (13.9)	348.4 (66.4)	174.5 (37.8)	1165.2 (38.7)	236.6 (49.1)	1.01 (0.2)	0.90 (0.1)
11–12	111.9 (12.3)	32.7 (8.17)	337.6 (66.8)	179.2 (35.7)	1161.5 (32.1)	215.1 (33.5)	0.94 (0.1)	0.88 (0.1)
9–10	111.3 (14.2)	32 (8.95)	335.5 (67.2)	175.1 (37.6)	1156.8 (35.2)	213.9 (43.5)	0.95 (0.2)	0.86 (0.1)
7–8	113.8 (13.7)	33.6 (11)	334.1 (70.8)	178.5 (40.3)	1152.8 (34.7)	216.6 (35)	0.95 (0.1)	0.87 (0.1)
5–6	112.3 (16.4)	35.7 (13.4)	316.7 (64.6)	168.1 (38.9)	1141.1 (38)	211.9 (46.3)	0.97 (0.2)	0.87 (0.1)
3–4	113.3 (16.1)	34.8 (12.4)	340.1 (73.4)	175.6 (43.8)	1146.9 (38)	213.5 (41)	0.96 (0.2)	0.88 (0.1)
1–2	114.1 (17.4)	36.4 (14.3)	334.6 (81.9)	177.9 (51.4)	1144 (45.7)	212.6 (48.7)	0.94 (0.2)	0.88 (0.1)
<1	111.4 (13.1)	35.1 (9.44)	326.4 (66.2)	170.3 (39.7)	1145 (39)	204.4 (39.9)	0.95 (0.2)	0.86 (0.1)
Never used HRT	111.7 (16.4)	36.3 (13.2)	323.7 (66.7)	167.5 (40.2)	1146.7 (38.6)	207 (43.4)	0.92 (0.2)	0.86 (0.1)
*P* value for trend^a^	.1	.03	.07	.07	.001	<.001	.002	.1
Last HRT use								
In the last year	115 (15.2)	33.1 (12)	354.1 (65.5)	175.7 (35)	1165.8 (33.8)	226.1 (45.6)	1.01 (0.1)	0.9 (0.1)
1–5 y ago	114.1 (14.6)	33.9 (10.9)	343.8 (69.8)	174.4 (41.7)	1152.2 (34.6)	217.2 (46.4)	0.94 (0.2)	0.85 (0.1)
>5 y ago	112.6 (15.5)	35 (12.1)	326.9 (70.9)	174.8 (42.9)	1146.5 (39.8)	211.5 (41.5)	0.95 (0.2)	0.87 (0.1)
Never used HRT	111.7 (16.4)	36.3 (13.2)	323.7 (66.7)	167.5 (40.2)	1146.7 (38.6)	207 (43.4)	0.92 (0.2)	0.86 (0.1)
*P* value (category)^a^	.4	.2	.01	.2	.007	.02	.001	.1

a Tests for trend or categories were based on regression models in which bone outcomes were logged, and age at period cessation and length of reproductive life and length of HT use were included as months since birth.

Length of HRT use was associated with lower medullary CSA and was strongly and positively related to polar SSI, cortical vBMD, and lumbar spine aBMD; associations with total and trabecular vBMD and total hip aBMD were weaker (Table 2). Recent use of HRT was also associated with polar SSI, cortical vBMD and total vBMD, and spine aBMD. There were no associations with bone CSA at any site.

### Differences in bone outcomes per 10-year difference in timing of period cessation (natural or surgical) and length of reproductive life

Women who had a later natural menopause had a 8.2% (95% confidence interval [CI] 1.3%–15.1%, *P* = .02) greater trabecular vBMD and a 6.0% (95% CI 0.5%–11.5%, *P* = .03) greater total vBMD than women with an age of menopause 10 years earlier (Table 3, model 1). There were no associations with age at hysterectomy (*P* value for interaction between menopause type and age at period cessation was .09 for trabecular vBMD and .02 for total vBMD). Similar-sized estimates were seen for the larger sample with spine and total hip aBMD. Adjustments for current height and weight (Table 3, model 2), adult occupation, and smoking had little effect on any of these estimates. Women who had a hysterectomy had better BMD than women who had a natural menopause (see Supplemental Figure 1, A and B); the interaction with age at period cessation meant that the differences were stronger in women with a younger age at cessation. They also had greater SSI (*P* = .05). There were no associations between age at natural menopause or age at hysterectomy and bone size or strength. The findings for length of reproductive life were similar (Supplemental Table 1). There was no evidence that the findings for hysterectomy status differed by oophorectomy status.

**Table 3.** Percentage Difference in Bone Outcomes per 10 Years of Difference in Timing of Period Cessation (Natural/Surgical), Adjusted for Type of Menopause, and Then Additionally Adjusted for Current Height and Weight

	Model 1			Model 2		
	Adjusted for Type of Menopause			Model 1 + Adjusted for Current Height and Weight		
	Difference, %	95% CI	*P* Value	Difference, %	95% CI	*P* Value
Diaphysis CSA (n = 562)						
Age at natural menopause	2	−1.6 to 5.7	.3	0.8	−2.5 to 4.1	.6
Age at hysterectomy	−1.3	−4.6 to 2.1	.5	−0.2	−3.2 to 2.8	.9
Medullary CSA (n = 561)						
Age at natural menopause	−4.8	−13.9 to 4.4	.3	−6.1	−15.1 to 2.8	.2
Age at hysterectomy	1.7	−6.7 to 10.1	.7	3	−5.2 to 11.3	.5
Total vBMD (n = 555)						
Age at natural menopause	6	0.5–11.5	.03	5.9	0.5–11.4	.03
Age at hysterectomy	0.8	−4.2 to 5.8	.8	0.7	−4.3 to 5.6	.8
Trabecular vBMD (n = 554)						
Age at natural menopause	8.2	1.3–15.1	.02	8.2	1.4–15.0	.02
Age at hysterectomy	0.1	−6.2 to 6.4	>.9	−0.2	−6.4 to 6.1	>.9
Cortical vBMD (n = 563)						
Age at natural menopause	0.5	−0.4 to 1.4	.3	0.5	−0.4 to 1.4	.3
Age at hysterectomy	−0.2	−1.1 to 0.6	.6	−0.2	−1 to 0.6	.6
Polar SSI (n = 563)						
Age at natural menopause	3.7	−1.7 to 9.1	.2	2.1	−2.8 to 6.9	.4
Age at hysterectomy	−2.8	−7.7 to 2.1	.23	−1.4	−5. to 3.1	.5
Lumbar spine aBMD (n = 703)						
Age at natural menopause	9.3	5.3–13.3	<.001	8.8	5.1–12.6	<.001
Age at hysterectomy	1.8	−2.0 to 5.6	.4	2.29	−1.3 to 5.8	.2
Total hip aBMD (n = 700)						
Age at natural menopause	6.7	3.2–10.3	<.001	6.4	3.4–9.4	<.001
Age at hysterectomy	−0.04	−3.3 to 3.2	>.9	0.4	−2.4 to 3.2	.8

### Differences in bone outcomes per 10-year HRT use and by time since last use

Length of HRT (Table 4) and recency of HRT use (Supplemental Table 2) were associated with greater SSI, higher cortical and total and trabecular vBMD, and greater aBMD, particularly in the lumbar spine; in some cases the estimates strengthened after adjusting for menopausal type (Table 4, model 2) and current height and weight (Table 4, model 3). For example, in the adjusted model, a 10-year difference in HRT use was associated with a 6.3% (95% CI 3.1%–9.4%, *P* < .001) greater polar SSI and a 0.9% (95% CI 0.3%–1.5%, *P* = .002) greater cortical vBMD. Further adjustment (data not shown) for smoking and adult occupation did not change these estimates. The association between length of HRT use and spine aBMD differed by type of menopause (*P* value for the interaction = .02), in that the association was less pronounced in those who had a hysterectomy compared with those with a natural menopause (Table 4 and Supplemental Table 2).

**Table 4.** Percentage Difference in Bone Outcomes per 10 Years of HRT Use, Unadjusted, and Then Adjusted for Type of Menopause and Additionally Adjusted for Current Height (Meters) and Weight (Kilograms)

	Unadjusted			Model 2			Model 3		
				Adjusted for Type of Menopause			Adjusted for Type of Menopause, Height and Weight		
	Difference, %	95% CI	*P* Value	Difference, %	95% CI	*P* Value	Difference, %	95% CI	*P* Value
Diaphysis CSA (n = 603)	1.9	−0.4 to 4.2	.1	1.8	−0.6 to 4.2	.1	2.4	0.3–4.5	.03
Medullary CSA (n = 602)	−6.5	−12.2 to −0.7	.03	−5.7	−11.7 to 0.2	.06	−5.5	−11.4 to 0.4	.07
Total vBMD (n = 597)	3.3	−0.3 to 6.8	.07	2.3	−1.3 to 6	.2	3.0	−0.6 to 6.7	.1
Trabecular vBMD (n = 596)	4.0	−0.3 to 8.3	.07	2.9	−1.6 to 7.3	.2	3.8	−0.6 to 8.2	.09
Cortical vBMD (n = 604)	0.9	0.4–1.5	.001	0.9	0.3–1.5	.003	0.9	0.3–1.5	.002
Polar SSI (n = 604)	6.0	2.6–9.3	<.001	5.4	1.9–8.9	.003	6.3	3.1–9.4	<.001
Spine L1-L4 aBMD (n = 747)	4.0	1.4–6.5	.002						
Ceased naturally				5.3	2.1–8.5	.001	6.7	3.7–9.7	<.001
Ceased surgically				−1.4	−6.1 to 3.2	.5	0.6	−3.9 to 5.0	.8
Hip aBMD (n = 742)	1.8	−0.4 to 3.9	.1	0.7	−1.5 to 3	.5	2.8	0.8–4.7	.007

*P* = .02 for the interaction between type of menopause and length of HRT.

### Differences in bone outcomes per 10-year difference in timing of period cessation (natural or surgical) or length of reproductive life, additionally adjusted for HRT use

After additional adjustment for length of HRT use, women with a later natural menopause still had greater trabecular vBMD and aBMD (Table 5 and Supplemental Table 3). Length of HRT use and recent HRT use remained positively associated with SSI, cortical vBMD, and aBMD, particularly of the lumbar spine. Similar results were seen for length of reproductive life (Supplemental Tables 4 and 5). Women who had a hysterectomy still had higher BMD after these adjustments than women who had a natural menopause. The interaction between menopause type and HRT use on lumbar spine was weaker (*P* > .1) than in the models in Table 4 (and Supplemental Table 2).

**Table 5.** Percentage Difference in Bone Outcomes by Type of Menopause per 10 Year Difference in Timing of Period Cessation (Natural or Surgical), per 10-Year Difference in HRT Use, Height, Weight, Smoking, and Adult Occupation

	Diaphysis CSA (n = 508)^a^			Medullary CSA (n = 507)^a^			Total vBMD, mg/cm^3^ (n = 502)^a^			Trabecular vBMD, mg/cm^3^ (n = 501)^a^		
	Difference, %	95% CI	*P* Value	Difference, %	95% CI	*P* Value	Difference, %	95% CI	*P* value	Difference, %	95% CI	*P* Value
Hysterectomy vs natural menopause (at age 50 y)	0.5	−2.7 to 3.7	.8	−3.1	−11.8 to 5.6	.5	5.8	0.5–11	.03	5.8	−0.8 to 12.4	.08
Age at period cessation, per 10 y												
Ceased naturally	0.1	−3.4 to 3.5	>.9	−8.5	−17.9 to 0.9	.08	3.7	−2 to 9.4	.2	7.1	0.04–14.2	.05
Ceased surgically	−0.3	−3.6 to 3	.8	0.5	−8.4 to 9.4	.9	2.4	−3 to 7.8	.4	1.5	−5.2 to 8.2	.7
HRT use, per 10 y	2.3	−0.3 to 4.9	.08	−6.3	−13.4 to 0.8	.08	2.5	−1.8 to 6.8	.3	4.0	−1.4 to 9.3	.1
Height, per 1 SD)	5.1	3.9 to 6.2	<.001	7.5	4.5 to 10.5	<.001	−2.5	−4.3 to −0.7	.008	−4.0	−6.3 to −1.8	.001
Weight, per 1 SD)	2.9	1.8 to 4.1	<.001	0.3	−2.9 to 3.4	.9	4.2	2.3–6.1	<.001	5.7	3.3 to 8	<.001
Smoking vs not smoking	1.1	−2.7 to 4.9	.6	2.1	−8.2 to 12.4	.7	0.7	−5.6 to 7	.8	−1.2	−9 to 6.7	.8
Manual vs nonmanual social class	−0.5	−3.4 to 2.3	.7	1.6	−6.1 to 9.3	.7	−0.6	−5.4 to 4.1	.8	0.9	−4.9 to 6.7	.8

	Cortical vBMD (n = 509)^a^			Polar SSI (n = 509)^a^			Lumbar Spine aBMD (n = 635)^a^			Total Hip aBMD (n = 632)^a^		
	Difference, %	95% CI	*P* Value	Difference, %	95% CI	*P* Value	Difference, %	95% CI	*P* Value	Difference, %	95% CI	*P* Value
Hysterectomy vs natural menopause	0.2	−0.7 to 1	.7	2.9	−1.8 to 7.6	.2	6.0	2.3 to 9.8	.002	3.7	0.8 to 6.7	.01
Age at period cessation, per 10 y												
Ceased naturally	0.5	−0.4 to 1.4	.3	1.0	−4.1 to 6.1	.7	8.8	4.8 to 12.7	<.001	5.8	2.6 to 8.9	<.001
Ceased surgically	0.2	−0.7 to 1.1	.6	−0.1	−4.9 to 4.8	>.9	3.3	−0.5 to 7.1	.09	1.6	−1.4 to 4.6	.3
HRT use, per 10 y	0.9	0.2 to 1.6	.01	6.3	2.5 to 10.2	.001	3.6	0.5 to 6.6	.02	2.7	0.3 to 5.1	.03
Height, per 1 SD	−0.01	−0.3 to 0.3	>.9	6.7	5.1 to 8.4	<.001	0.7	−0.6 to 2	.3	−0.4	−1.4 to 0.7	.5
Weight, per 1 SD	0.3	−0.03 to 0.6	.07	4.5	2.8 to 6.2	<.001	6.2	4.9 to 7.5	<.001	8.1	7.1 to 9.2	<.001
Smoking vs not smoking	−0.2	−1.3 to 0.8	.6	1.0	−4.6 to 6.6	.7	−2.2	−6.7 to 2.3	.3	−3.2	−6.9 to 0.4	.09
Manual vs nonmanual social class	−0.3	−1.1 to 0.4	.4	−3.2	−7.4 to 1	.1	−0.6	−3.9 to 2.6	.7	−1.1	−3.7 to 1.5	.4

a Samples exclude between 53 and 68 women with insufficient data on HRT use.

## Discussion

We have shown in a large British cohort of women that a 10-year later age at natural menopause was associated with an estimated 6%–8% greater trabecular vBMD in women aged 60–64 years, even after adjusting for body size, HRT use, and social and behavioral factors. A longer length of reproductive life showed similar consistent and positive associations with the same bone parameters. HRT use was associated with a 0.9% greater cortical vBMD, 6.3% smaller medullary CSA, and a 6% greater SSI; the associations with total and trabecular vBMD were weaker. Age at natural menopause, length of reproductive life, and HRT use in women who had a natural menopause were also associated with aBMD of the lumbar spine and total hip.

### Comparison with other studies and interpretation

#### Natural menopause, length of reproductive life, and bone

Previous studies have shown that an earlier natural menopause and a shorter reproductive life are associated with lower BMD (2, 4, 6, 8). Using transilial biopsy specimens, Akhter et al (28) observed that across the menopause transition, there was decreasing bone tissue volume to total volume and trabecular number and increased trabecular spacing, which would explain the changes in microarchitecture detected as lower BMD using DXA or pQCT. Our findings show that the inverse associations with early menopause and shorter reproductive life persist into the seventh decade of life and are observed for vBMD and aBMD. Whether these effects will eventually be attenuated by age as a risk factor for fracture and so have little long-term effect on hip fracture risk, as indicated in the Million Women Study (16), cannot yet be determined, but our study has one of the longest follow-up periods to date.

There is a need to separate age- and menopause-related mechanisms that affect bone health. Although the loss of BMD is initially in the trabecular compartment and in women is most strongly related to menopause, it is followed by an equivalent decline in cortical vBMD because endo- and intracortical resorption accelerates and periosteal expansion slows, leading to a reduction in cortical area, and consequently in bone strength (29). Factors other than declining sex hormones may play a greater role in this aspect of bone loss and may explain why the associations between natural menopause and reproductive life and bone differed from the associations evident for HRT use.

Our finding that a shorter reproductive life was associated with lower BMD suggests that lifetime cumulative estrogen exposure may be important. In determining the duration of endogenous estrogens, Hagemans et al (30) concluded that knowing age at menarche and menopause was sufficient; having information on parity, miscarriages, lactation, oral contraceptive use, and length of menstrual cycle did not explain any further variation in BMD, adding strength to the observations in the current study. Various other factors that we are unable to study will contribute to menopausal bone loss such as declining levels of estradiol and FSH (31), cytokines (32), genetic factors (33), and bone, muscle, and fat interactions (34).

### HRT use and bone

Randomized control trials have shown increased aBMD in hip and lumbar spine and protection from fracture in HRT users (13, 14, 35–39). Our finding of greater aBMD for HRT users, particularly in the lumbar spine, a site containing mostly trabecular bone, is consistent with these findings,

Findings from our study of the short-term benefits of HRT on bone are likely to be due to the mechanism by which it acts: increased cortical vBMD and a narrower medullary cavity are likely to be due to reductions in both intracortical remodeling and endocortical resportion, both of which would increase bone strength. The findings support the view that HRT protects cortical bone from age-related changes in endocortical resorption and reduced bone turnover. Previous smaller studies have also shown that HRT users compared with nonusers have higher vBMD, larger cortical CSA, and greater bending and compressive bone strength in the tibial shaft, a weight-bearing site, as well as the distal radius (40, 41), which is consistent with our findings. A small longitudinal study of HRT users compared with a control group suggested that exogenous estrogen fills the small marrow pores close to the endocortical surface so that the pQCT-defined boundary between trabecular and cortical bone shifted in favor of cortical bone, conferring greater strength to the bone (22). Mikkola et al (18) carried out a long-term follow-up of monozygotic twin pairs and showed greater cortical and trabecular vBMD at distal and diaphyseal sites in the twin taking HRT compared with the other twin who was not; these differences resulted in greater compressive and bending strength. They suggested that HRT may become more important with years from menopause as the study showed an annual increase of 2.6%–2.8% in intrapair difference in bone strength. Given the results of these two studies, it was surprising that we did not find an effect of HRT on trabecular or total BMD at the distal radius. This may be due to limitations in the spatial resolution of pQCT, meaning we could not accurately define the cortical, subcortical, and trabecular boundaries and so detect differences in the bone compartments.

### Hysterectomy status and bone

Women in this cohort who had a hysterectomy had greater BMD than women with a natural menopause. The difference was greatest for women who had an earlier age at period cessation. It was reduced in the models that included all women in whom use of HRT was known, suggesting that longer use of HRT contributed to greater BMD in women with a hysterectomy. These women were also of heavier weight, and previous NSHD studies have shown they were also more likely to be overweight or obese in midlife and have an earlier menarche (42, 43). So these factors too may partly explain the association. The most common reason for a hysterectomy, particularly at earlier ages, was fibroids (43), which may have been associated with greater estrogen exposure through earlier menarche, and contributed to greater BMD. There is little evidence from other studies that hysterectomy or oophorectomy is associated with bone outcomes or fracture risk (9–12), although few studies have examined the reasons for the operations, which may be of consequence (11).

### Strengths and limitations

The main strengths of this study are the prospective, detailed, and longitudinal collection of data on menopausal characteristics in relation to gold standard bone outcomes on a relatively large sample of British women followed up into early old age. pQCT and DXA measurements were obtained; pQCT enables the investigation of bone size, strength, and vBMD of trabecular and cortical compartments with less confounding by body size, which is a limitation of aBMD obtained by DXA. That all the women were born in the same week, and that the scans took place over a narrow age range of 10 years after the average age at menopause, limited potential confounding by age and enabled an assessment of the persistence of menopause-related effects on bone. It also allowed the study of how HRT use may protect from fracture through slowing down age-related changes in vBMD and endocortical resportion that decrease bone strength.

A limitation is that we did not collect HRT dose, and data on types of HRT preparations were insufficiently complete to use. We have previously reported that the vast majority on HRT who had had a hysterectomy were taking estrogen alone, whereas other women were taking a combined preparation (44). Data on length of use and age at last use were advantages over studies that have only collected measures of current and past HRT use. Another limitation is that the sample was all born in the early postwar period; our findings may not be generalizable to later born cohorts. Whereas these cohorts have experienced little change in the timing of natural menopause, HRT use has declined since the adverse reports from clinical trials, and there has been a small decline in pubertal timing (45). HRT use in this cohort showed a distinct drop during 2002 (age 56 y) at the time of adverse trial reports (46). In this context, HRT prescriptions for participants whose periods ceased from this time (who were more likely to have greater BMD) were less likely, whereas HRT may still have been prescribed to women seen to be at high risk of fracture (including those with early period cessation). Thus, the associations between HRT use and BMD could have been weakened.

In conclusion, this study shows that later natural menopause and longer reproductive life are associated with greater trabecular vBMD and aBMD in early old age and that HRT use is associated with greater cortical vBMD, bone strength, and aBMD. Although HRT is not likely to be restored as an agent for common use primarily for the prevention of osteoporosis, this study showed protective effects on bone for women with natural menopause taking the therapy.
